# Digitale Pathologie

**DOI:** 10.1007/s00292-024-01306-9

**Published:** 2024-02-28

**Authors:** Nadine Flinner, Peter Boor, Peter J. Wild

**Affiliations:** 1 Dr. Senckenbergisches Institut für Pathologie, Universitätsklinikum Frankfurt, Goethe-Universität Frankfurt, Theodor-Stern-Kai 7, 60590 Frankfurt am Main, Deutschland; 2 Frankfurt Institute for Advanced Studies (FIAS), Frankfurt am Main, Deutschland; 3 Frankfurt Cancer Institute (FCI), Frankfurt am Main, Deutschland; 4 University Cancer Center (UCT), Frankfurt-Marburg, Deutschland; 5 Institut für Pathologie, Sektion Nephropathologie, Universitätsklinikum RWTH Aachen, Aachen, Deutschland; 6 Medizinische Klinik II, Universitätsklinikum RWTH Aachen, Aachen, Deutschland; 7 Wildlab, University Hospital Frankfurt MVZ GmbH, Frankfurt am Main, Deutschland

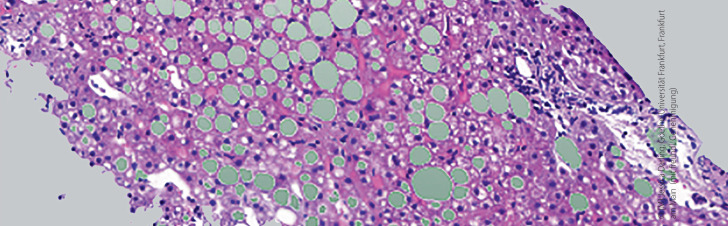



Liebe Kolleg/innen,

die heutige Ausgabe von *Die Pathologie* beschäftigt sich mit dem Thema digitale Pathologie. Damit haben wir ein Thema gewählt, das im Arbeitsalltag immer mehr Bedeutung gewinnt und noch mehr gewinnen wird.

Ein wichtiger erster Schritt der digitalen Pathologie ist die Digitalisierung von Schnittpräparaten durch sog. Slidescanner, womit sog. Whole Slide Images (WSIs) entstehen. Dies bringt einige Vorteile mit sich, wie z. B. die Möglichkeit, digitale Bildanalysen durchzuführen, die Nutzung digitaler Schnitte für Tumorboards und wissenschaftliche Zwecke oder das mobile Arbeiten im Homeoffice. Leider sind hiermit auch Nachteile verbunden. So müssen z. B. geeignete Arbeitsplätze für Pathologen (Monitore, Slide Viewer etc.) sowie ein Image-Management-System (IMS) zum Bereitstellen der Dateien (redundant, ausfallsicher etc.) beschafft werden, wobei vor allem die Speicherkosten (> 1 GB pro WSI) berücksichtigt werden müssen. Das nachfolgende Themenheft gibt einen Überblick über sämtliche Themenfelder einer digitalen Transformation in der Pathologie. Methoden künstlicher Intelligenz werden in gleicher Weise vorgestellt wie personelle und rein technische Aspekte im Labor.

Der erste Artikel von Schwaibold et al. zeigt eindrucksvoll, wie bauliche Veränderungen im Labor, die eine bessere Reinigung der Arbeitsplätze ermöglichen, den benötigten Speicherplatz signifikant reduzieren, da sich weniger fremde Gewebepartikel auf den Schnitten befinden.

Der zweite Artikel von Iwuajoku et al. beschreibt die „Dos und Don’ts“, wenn in der Routinediagnostik alle Schnitte gescannt werden. Die Autoren identifizieren 3 Probleme, die hierbei entstehen: technische Probleme mit Scannern (z. B. nichterkannte Racks, Netzwerkunterbrechungen), Fehler im Betriebsablauf (z. B. schlecht geschultes Personal) und geringe Qualität der Objektträger (z. B. falsch aufgebrachte Barcodes/Deckgläser).

Die Computational Pathology befasst sich mit der Analyse der digitalen Schnitte. Es werden Algorithmen entwickelt, die z. B. Vorhersagen zur Prognose oder dem Therapieansprechen generieren oder Objekte quantitativ erfassen können. Für die Entwicklung dieser Algorithmen ist es wichtig, dass Pathologen und IT-Experten eng zusammenarbeiten, was in 2 weiteren Artikeln demonstriert wird. Stoll et al. zeigen am Beispiel der Vorhersage von molekularen Subtypen beim muskelinvasiven Harnblasenkarzinom, dass Datensätze in öffentlichen Datenbanken nicht immer fehlerfrei sind und dass die Neuformulierung des zu lernenden Problems eine Verbesserung in der Performance bringen kann. Solche Algorithmen zur molekularen Subtypisierung könnten zur Minimierung von Kosten eingesetzt werden, wenn sie als Screeningtool verwendet werden, um medizinisch sinnvolle In-situ-Verfahren und molekulare Verfahren für jeden Patienten individuell vorauszuwählen. Bei der Quantifizierung von Fett in der Leber, z. B. im Rahmen der Beurteilung von Leberexplantaten für die Transplantation, beschreiben Darling et. al. das Problem, dass viele veröffentlichte Algorithmen nicht das Erfassen, was wirklich vom Pathologen beurteilt wird. Die Arbeit zeigt zudem, dass die Hinzunahme von zusätzlichen Informationen (z. B. Fibrose und Entzündung) tatsächlich zu verbesserten Algorithmen führen kann, die am Ende sogar das Potenzial haben, das Problem der Inter- und Intraobservervariabilität zu minimieren.

Wenn solche Algorithmen wirklich in der Routine verwendet werden sollen, dann müssen diese nicht nur auf den Entwicklungsdaten eine ausgezeichnete Performance aufweisen, sondern müssen auch auf den Datensätzen des eigenen Instituts performant sein; also übertragbar und erklärbar sein. Mayer et al. fassen hier die wichtigsten Faktoren zusammen, die beachtet werden müssen, wenn KI-Modelle trainiert werden, und stellt Methoden vor, die helfen können, diese Transferierbarkeit von Algorithmen zu verbessern. Ein weiterer wichtiger Punkt neben der Übertragbarkeit ist die Erklärbarkeit der KI-Vorhersagen („explainable articial intelligence“, XAI), um diese für den Pathologen verifizierbar und nachvollziehbar zu machen. Der aktuelle Stand ist im Artikel von Klauschen et al. zusammengefasst und zeigt außerdem, wie die gleichen Methoden in der Forschung genutzt werden können, um Biomarker zu identifizieren. Neben Transferierbarkeit und XAI ist auch die „trustworthy AI“ ein wichtiger Forschungsbereich, in dem es darum geht, Algorithmen so zu entwickeln, dass die Unsicherheit quantitativ erfasst wird und somit anstelle einer möglicherweise falschen Vorhersage keine Ausgabe generiert wird.

Unser letzter Artikel von Bülow et al. führt den Begriff der Pathomics ein. Hierbei handelt es sich um eine Methode, die quantitative Messungen aller relevanten, histologischen Objekte eines Schnittes automatisch, reproduzierbar und standardisiert erfasst. Der Artikel zeigt auf, wie Pathomics für die Nephropathologie genutzt werden kann und letztlich die Pathologie von einer qualitativen und beschreibenden hin zu einer quantitativen Disziplin werden lassen kann.

Wir sind überzeugt, dass die Digitalisierung der Pathologie viele Vorteile für Patienten und Pathologen bringen wird, auch wenn die initialen Investitionskosten in die Infrastruktur hoch sind. Die meisten Pathologien in Deutschland arbeiten noch nicht vollständig digital, verfügen jedoch bereits über Slidescanner. Die Digitalisierung des Workflows schreitet jedoch zügig voran. Bund und Länder haben hier, z. B. durch das Krankenhauszukunftsgesetz (KHZG), einen Anfang gemacht. Die digitale Transformation wird das Fach Pathologie und die Arbeitsweise in Zukunft nachhaltig verändern. Es liegt an uns allen, diese Veränderung proaktiv mitzugestalten.

Wir wünschen Ihnen viel Spaß beim Lesen und Durchblättern!

Peter Boor, Nadine Flinner und Peter J. Wild

